# Cardiff Showing the Way

**Published:** 1920-01-31

**Authors:** 


### Cardiff showing the Way.

WE have received a letter from Cardiff containing thanks and acknowledgment for the stimulating article last week. We certainly do think that Cardiff has risen to the occasion and is setting an example to all. The gift of the ex-Service men, reported in another column, is a jewel of self-sacrifice and generosity that will be memorable among hospital gifts. Our sympathy goes out to the Lord Mayor, who is exhibiting the power of personality and personal effort in a splendid cause. He has the zealous, able, and continuous support of Mr. Leonard D. Rea, who is at work for his hospital continuously, and has earned and should receive prompt and adequate relief and assistance without a day's delay. Then King Edward VII. Hospital, Cardiff, would gain much and secure results which must be impossible so long as Mr. Rea is so overborne as to resemble at times the toad, or shall we say the angel of leading, under the harrow.

